# MiR-155-mediated loss of C/EBPβ shifts the TGF-β response from growth inhibition to epithelial-mesenchymal transition, invasion and metastasis in breast cancer

**DOI:** 10.1038/onc.2013.322

**Published:** 2013-08-19

**Authors:** J Johansson, T Berg, E Kurzejamska, M-F Pang, V Tabor, M Jansson, P Roswall, K Pietras, M Sund, P Religa, J Fuxe

**Affiliations:** 1 Division of Vascular Biology, Department of Medical Biochemistry and Biophysics, Karolinska Institute, Stockholm, Sweden; 2 Department of Medicine, Centre for Molecular Medicine, Karolinska Institute, Stockholm, Sweden; 3 Department of Internal Medicine, Medical University of Warsaw, Warsaw, Poland; 4 Department of Surgical and Perioperative Sciences, Surgery, Umeå University, Umeå, Sweden; 5 Department of Laboratory Medicine Malmö, Lund University Cancer Center, Lund University, Malmö, Sweden

**Keywords:** CCAAT-enhancer binding protein beta, epithelial-mesenchymal transition, transforming growth factor-beta, Breast cancer, metastasis

## Abstract

During breast cancer progression, transforming growth factor-beta (TGF-β) switches from acting as a growth inhibitor to become a major promoter of epithelial-mesenchymal transition (EMT), invasion and metastasis. However, the mechanisms involved in this switch are not clear. We found that loss of CCAAT-enhancer binding protein beta (C/EBPβ), a differentiation factor for the mammary epithelium, was associated with signs of EMT in triple-negative human breast cancer, and in invasive areas of mammary tumors in MMTV-PyMT mice. Using an established model of TGF-β-induced EMT in mouse mammary gland epithelial cells, we discovered that C/EBPβ was repressed during EMT by miR-155, an oncomiR in breast cancer. Depletion of C/EBPβ potentiated the TGF-β response towards EMT, and contributed to evasion of the growth inhibitory response to TGF-β. Furthermore, loss of C/EBPβ enhanced invasion and metastatic dissemination of the mouse mammary tumor cells to the lungs after subcutaneous injection into mice. The mechanism by which loss of C/EBPβ promoted the TGF-β response towards EMT, invasion and metastasis, was traced to a previously uncharacterized role of C/EBPβ as a transcriptional activator of genes encoding the epithelial junction proteins E-cadherin and coxsackie virus and adenovirus receptor. The results identify miR-155-mediated loss of C/EBPβ as a mechanism, which promotes breast cancer progression by shifting the TGF-β response from growth inhibition to EMT, invasion and metastasis.

## Introduction

Breast cancer progression is associated with several hallmarks of cancer, such as loss of epithelial differentiation, and evasion of growth inhibitory and apoptotic cell responses.^1^ Transforming growth factor-beta (TGF-β) is at the center of these hallmarks, as it is a prominent growth inhibitor of normal and pre-malignant epithelial cells, but switches to become a major promoter of invasion and metastasis during breast cancer progression.^2, 3, 4^ Thus, TGF-β has dual roles in breast cancer by acting as a tumor suppressor in early stages, and a promoter of invasion and metastasis in advanced disease. Understanding the molecular mechanisms of this switch in the TGF-β response has remained an elusive goal in cancer research.

TGF-β promotes tumor cell invasion and metastasis by inducing epithelial-mesenchymal transition (EMT), a latent developmental process, which is reactivated in cancer tissues.^5, 6^ During EMT, genes encoding epithelial proteins, such as the junction proteins E-cadherin and coxsackie virus and adenovirus receptor (CAR) are inactivated. In parallel, genes encoding mesenchymal proteins, such as vimentin and N-cadherin are induced. As a result, tumor cells undergoing EMT gain the capacity to detach and migrate into the surrounding tissue. Thus, EMT converts benign tumor cells into malignant counterparts by inducing a de-differentiation program. EMT is specifically linked to triple-negative breast cancers, which have lost expression of the estrogen receptor, the progesterone receptor and the human epidermal growth factor receptor (HER2).^7, 8^ Furthermore, EMT promotes the acquisition of stem cell characteristics suggesting that cancer stem cells may originate from more differentiated tumor cells undergoing EMT.^9^

TGF-β signaling towards EMT involves activation of Smad3/4 transcription factors, which form EMT-promoting complexes with cofactors, such as Snail, Zeb, AP-1 and NF-KB.^3, 10, 11, 12^ TGF-β signaling towards growth inhibition is mediated by Smad complexes with FoxO factors, which activate the cell cycle inhibitors p15INK4b and p21CIP1,^13, 14^ and with E2F4/5 and ATF3 factors, which inactivate c-Myc and ID1, respectively.^15, 16, 17^ In addition, the transcription factor CCAAT-enhancer binding protein beta (C/EBPβ) was identified as a component of these complexes, which is essential both for the induction of p15INK4b and the repression of c-Myc, and therefore central to the cytostatic program initiated by TGF-β.^18^ Further studies have shown that C/EBPβ-mediated growth inhibition is averted in human epidermal growth factor receptor 2 (HER-2)-overexpressing breast cancer cells via an increased ratio of liver inhibitory protein (LIP) versus liver activating protein (LAP) and thereby inhibition of the transcriptional activity of LAP.^19^

C/EBPβ is a differentiation factor for the mammary epithelium and is produced, through alternative initiation of translation, in three isoforms: the transcriptional activators liver activating protein 1 and 2 (LAP1 and LAP2), and the LIP, which can inhibit C/EBPβ-mediated gene activation.^20, 21, 22^ C/EBPβ has been reported to be deregulated in breast cancer but its role in cancer progression is not fully elucidated (reviewed in Zahnow^22^). Loss of the transcriptional activation function of LAP due to an increased LIP/LAP ratio was identified as a mechanism of evasion of the cytostatic response to TGF-β in metastatic breast cancer cells isolated from pleural effusions.^18, 23, 24^ The mechanism was traced to a role of C/EBPβ as a transcriptional activator of *INK4b*, a gene encoding the cyclin-dependent kinase inhibitor p15INK4b, which is an important mediator of growth inhibition in response to TGF-β.^18^ These data show that loss of C/EBPβ-mediated gene activation promotes breast cancer progression. Interestingly, overexpression of LAP2 was shown to induce EMT-like features in cultured breast cancer cells, suggesting that C/EBPβ might be involved in the TGF-β response towards both growth inhibition and EMT.^25^

We hypothesized that de-regulation of C/EBPβ might be associated with TGF-β-induced EMT during breast cancer progression. To determine this, we combined studies of human breast cancer, mouse models of TGF-β-driven mammary cancer progression driven a model of TGF-β-induced EMT in mammary epithelial cells. The results indicate that the loss of C/EBPβ in breast cancer promotes malignant progression by shifting the TGF-β response from growth inhibition to EMT, invasion and metastasis.

## Results

### Loss of C/EBPβ marks signs of EMT in invasive breast cancer

**Figure 1:**
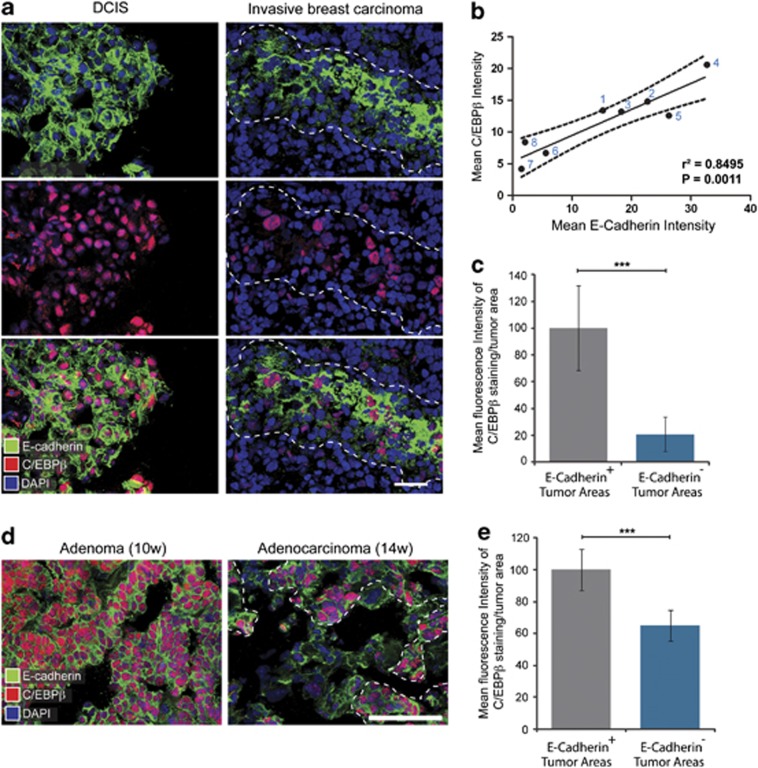
Loss of C/EBPβ is associated with EMT in invasive breast cancer. (**a**) Representative confocal microscopy images of samples of human ductal carcinoma *in situ* (DCIS) and invasive breast carcinoma stained by immunofluorescence with antibodies against E-cadherin and C/EBPβ. The outlined region of the invasive breast carcinoma marks an E-cadherin^+^ area of the tumor. Cell nuclei were labeled with DAPI. Scale bar, 50 μm. (**b)** Graph showing results from linear regression analysis of quantitative measurements of the fluorescence intensity of C/EBPβ (*y* axis) and E-cadherin (*x* axis) staining in eight different invasive breast tumors. The best-fit line is indicated as well as two confidence bands (dotted) that defined the confidence interval (95%). Tumors 6, 7 and 8 had been classified as triple-negative breast tumors (See Supplementary Figure 1 for characterization of the different tumors). (**c**) Bar graph showing results from measurements of mean fluorescent intensity of C/EBPβ staining in E-cadherin^+^ versus E-cadherin^−^ tumor areas of the three triple-negative tumors (#6, 7, 8). Mean fluorescent intensity values of C/EBPβ in E-cadherin^+^ areas were normalized to 100%. (**d**) Representative images showing immunofluorescence staining for E-cadherin and C/EBPβ in adenomas and adenocarcinomas of 10- and 14-week-old MMTV-PyMT mice, respectively. The outlined region marks an E-cadherin^+^ area of the tumor. Cell nuclei were labeled with DAPI. Scale bar, 50 μm. (**e**) Bar graph showing results from measurements of mean fluorescent intensity of C/EBPβ staining in E-cadherin^+^ versus E-cadherin^−^ tumor areas of adenocarcinomas from 14-week-old MMTV-mice. Mean fluorescent intensity values of C/EBPβ in E-cadherin^+^ areas were normalized to 100%. ***P<0.001.

Immunofluorescence staining and subsequent confocal microscopy analysis of samples of human breast carcinoma was performed to determine whether signs of EMT and loss of cell differentiation is associated with alterations in expression of C/EBPβ. As loss of E-cadherin is a hallmark of EMT in breast cancer, we co-stained for E-cadherin and C/EBPβ. Prominent staining of E-cadherin at cell–cell junctions and nuclear staining of C/EBPβ was detected in breast cancer cells in samples of well-differentiated ductal carcinoma *in situ* (DCIS) (Figure 1a). Furthermore, we analyzed the expression of E-cadherin and C/EBPβ in a series of eight invasive ductal breast carcinomas, which had been classified according to their status of estrogen receptor, progesterone receptor and HER2 (Supplementary Figure 1). We found decreased expression of E-cadherin in areas of triple-negative tumors (Figure 1a) and that these tumors (number 6, 7, 8) expressed decreased levels of E-cadherin compared with tumors that were positive for estrogen receptor, progesterone receptor and HER2, or only HER2 (Figure 1b). Nuclear staining for C/EBPβ was decreased in triple-negative tumors and linear regression analysis showed significant correlation (*P*=0.0011) between the expression of E-cadherin and C/EBPβ in the eight tumors analyzed (Figures 1a and b). Further analysis demonstrated significantly decreased expression of C/EBPβ in E-cadherin-negative areas compared with E-cadherin-positive areas of the triple-negative breast tumors (Figure 1c).

To study the expression of C/EBPβ during mammary cancer progression, we stained sections from transgenic mice overexpressing the polyoma virus middle T antigen under the mouse mammary tumor virus promoter (MMTV-PyMT mice). Female heterozygous MMTV-PyMT mice develop palpable hyperplastic lesions at around 4 weeks of age. These lesions develop into adenomas that become macroscopically visible and grow in size and at 10 weeks of age, they are classified as adenomas. However, at 12–14 weeks of age, the tumors switch on a more invasive phenotype and progress into adenocarcinomas that disseminate and form lung metastases. Metastatic spread of tumor cells in the MMTV-PyMT model is driven by TGF-β.^26^ Co-staining for E-cadherin and C/EBPβ in tumor sections from 10- and 14-week-old MMTV-PyMT mice revealed significant loss of C/EBPβ expression in E-cadherin-negative areas compared with E-cadherin-positive tumor areas of adenocarcinomas (Figures 1d and e).

### Loss of C/EBPβ during TGF-β1-induced EMT

**Figure 2:**
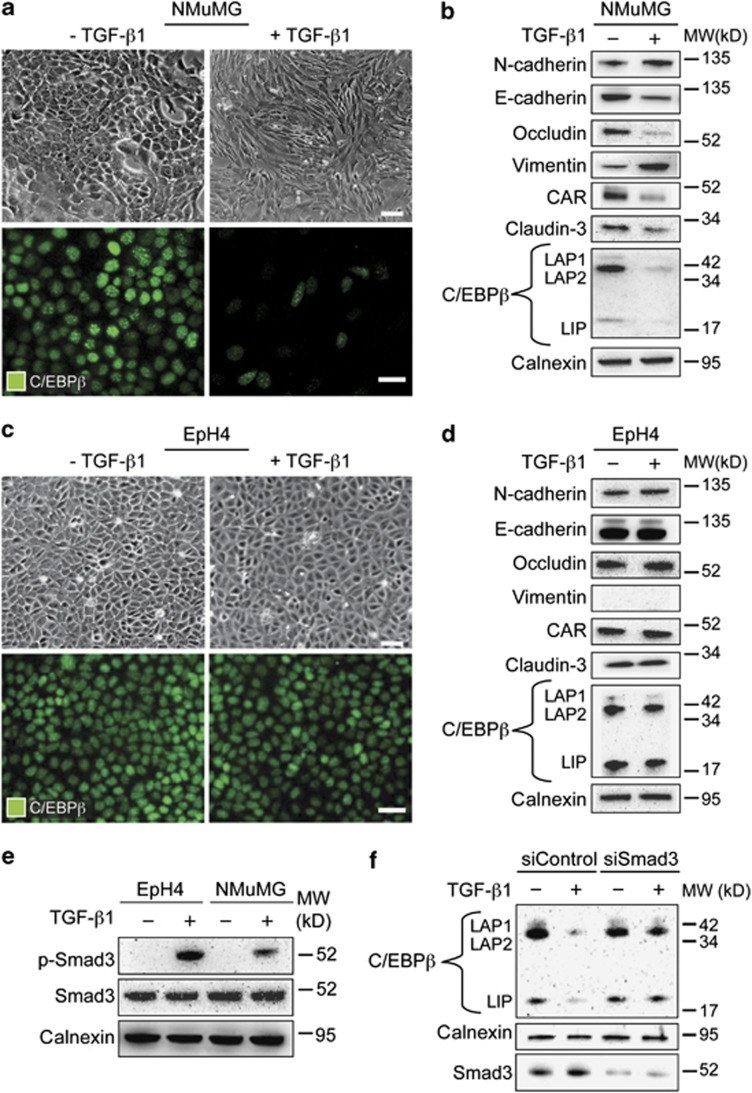
Loss of C/EBPβ is linked to the induction of EMT in response to TGF-β1. (**a**, **c**) Representative brightfield and immunofluorescence images showing induction of morphological characteristics of EMT and loss of nuclear staining of C/EBPβ in NMuMG cells (**a**) but not in EpH4 cells (**c**) after TGF-β1 treatment (10 ng/ml, 48 h). Scale bars, 50 μm. (**b**, **d**) Results from immunoblotting analysis showing changes in the expression of established EMT markers, and decreased expression of all three isoforms of C/EBPβ (LAP1, LAP2 and LIP) in NMuMG cells (**b**) but not in EpH4 cells (**d**) after TGF-β1 treatment (10 ng/ml, 48 h). (**e**) Immunoblotting analysis of the effect of TGF-β1 treatment (10 ng/ml, 1 h) on phosphorylation of Smad3 (p-Smad3) in NMuMG and EpH4 cells. (**f**) Immunoblotting analysis of the effect of siRNA-mediated knockdown of Smad3 (100 nM, 72 h) on the repression of C/EBPβ during TGF-β1-induced EMT (5 ng/ml, 24 h) in NMuMG cells. Calnexin was used as a loading control for all immunoblotting experiments.

Our results indicated that loss of C/EBPβ in breast cancer was linked to loss of E-cadherin, a hallmark of EMT, which prompted us to study whether C/EBPβ is regulated during EMT. We used an established model of TGF-β1-induced EMT in mouse mammary gland epithelial cells (NMuMG). As expected, treatment of NMuMG cells with TGF-β1 (10 ng/ml) for 48 h resulted in cellular elongation (Figure 2a), as well as loss of E-cadherin and induction of vimentin (Supplementary Figure 2a). Distinct nuclear staining of C/EBPβ was detected in untreated NMuMG cells, but was decreased in cells treated with TGF-β1 (Figure 2a). Immunoblotting analyses confirmed the EMT phenotype of TGF-β1-treated NMuMG cells that expressed decreased levels of the junction proteins E-cadherin, CAR, occludin and claudin-3, and increased levels of N-cadherin and vimentin, compared with untreated cells (Figure 2b). Immunoblotting analysis of C/EBPβ showed that all three C/EBPβ isoforms (LAP1, LAP2 and LIP) were decreased in TGF-β1-treated NMuMG cells (Figure 2b). No obvious changes in the ratio between LIP and LAP isoforms were detected.

To study whether loss of C/EBPβ was specifically coupled to an EMT response downstream of TGF-β1, we analyzed expression of C/EBPβ in mouse mammary EpH4 epithelial cells, which are known to be resistant to TGF-β1-induced EMT.^27, 28^ Treatment of EpH4 cells with TGF-β1 (10 ng/ml) for 48 h did not result in any morphological signs of EMT, nor any changes in the expression of junction proteins, or the mesenchymal proteins vimentin and N-cadherin (Figures 2c and d and Supplementary Figure 2b). However, activation of Smad3 was induced in EpH4 cells after TGF-β1 treatment (Figure 2e). C/EBPβ was prominently expressed in EpH4 cells and the relative level of LIP versus LAP2 was higher in EpH4 cells compared with NMuMG cells (Figure 2d). No changes in C/EBPβ levels were detected in EpH4 cells after TGF-β1 treatment.

To study whether the repression of C/EBPb was mediated through the canonical TGF-β/Smad3 pathway, we performed studies in which we inhibited the action of Smad3. Loss of C/EBPb during TGF-β1-induced EMT (5 ng/ml, 24 h) was inhibited by both siRNA-mediated knockdown of Smad3 (Figure 2f), and in the presence of a commercially available specific inhibitor of Smad3 (Supplementary Figure 2c).

The data indicated that loss of C/EBPβ is a result of a signaling event occurring downstream of receptor-Smad signaling and specifically associated with an EMT response to TGF-β1 in mammary epithelial cells. In further support of this, we found that all three isoforms of C/EBPβ were significantly less expressed in MDA-MB-231 cells, which are invasive human breast cancer cells that display EMT features, compared with MCF7 cells, which are non-invasive, non-EMT-like human breast cancer cells (Supplementary Figure 2d). Moreover, we found that all three C/EBPβ isoforms were decreased in another frequently used model of TGF-β1-induced EMT in human lung carcinoma A549 cells (Supplementary Figure 2e). This further linked loss of C/EBPβ to an EMT response and showed that this effect is not restricted to mammary epithelial cells.

### MiR-155-mediated depletion of C/EBPβ during TGF-β1-induced EMT

**Figure 3:**
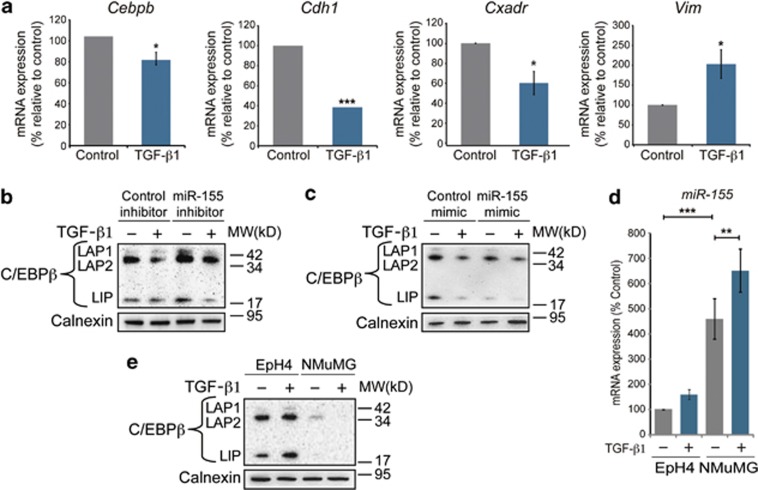
Depletion of C/EBPβ during TGF-β1-induced EMT by miR-155. (**a**) Results from qPCR analysis of the effect of TGF-β1 (10 ng/ml, 24 h) on mRNA levels of *Cebpb*, *Cdh1*, *Cxadr* and *Vim* relative to control, which was normalized to 100%. Data are mean ±s.e.m. of three independent experiments. (**b**, **c**) Immunoblotting analysis of the effect of a miR-155 inhibitor (**b**) and a miR-155 mimic (**c**) on the repression of C/EBPβ during TGF-β1-induced EMT (5 ng/ml, 24 h). (**d**) Results from qPCR analysis of the expression of miR-155 in EpH4 and NMuMG cells at baseline and after treatment with TGF-β1 (10 ng/ml, 24 h). (**e**) Immunoblotting analysis of the relative expression levels of C/EBPβ in EpH4 and NMuMG cells at baseline and after treatment with TGF-β1 (10 ng/ml, 48 h). Calnexin was used as a loading control for all immunoblotting experiments. *P<0.05; **P<0.01, ***P<0.001.

Next, we wanted to determine the mechanism of C/EBPβ repression during TGF-β1-induced EMT. Compared with messenger RNA (mRNA) levels of E-cadherin (*Cdh1*) and CAR (*Cxadr*), which were reduced by 60 and 40%, respectively, in NMUMG cells after TGF-β1 treatment (10 ng/ml, 24 h), mRNA levels of C/EBPβ (*Cebpb*) were less reduced (20%) (Figure 3a). This suggested that posttranscriptional mechanisms could be involved in repressing C/EBPβ during TGF-β1-induced EMT. Based on recent data showing that *Cebpb* is targeted by microRNA-155 (miR-155) in B cells and macrophages,^29, 30^ and the fact that miR-155 is a well-known oncomiR in breast cancer, we hypothesized that miR-155 could have a role in repressing C/EBPβ during TGF-β-induced EMT. We treated NMuMG cells with a commercially available synthetic miR-155 inhibitor (50 nM), which is specifically designed to target and irreversibly bind and inactivate miR-155, 2 h prior to TGF-β1 treatment (5 ng/ml, 24 h) to study whether the presence of the miR-155 inhibitor would affect the expression of C/EBPβ. We found that in the presence of the miR-155 inhibitor (50 nM), the expression of C/EBPβ was increased at baseline, and less repressed during EMT (Figure 3b). Immunofluorescence analysis further indicated that inhibition of miR-155 resulted in increased nuclear expression of C/EBPβ at baseline, and less repression during TGF-β1-induced EMT (Supplementary Figure S3a). No obvious rescue of the repression of E-cadherin or the induction of vimentin was observed neither in immunofluorescence (Supplementary Figure S3a), nor immunoblot analysis (data not shown). However, quantitative PCR (qPCR) analysis showed a 20% rescue of the repression of *Cdh1* mRNA during TGF-b-induced EMT (Supplementary Figure S3b). On the contrary, treatment of NMuMG cells with a synthetic miR-155 mimic (50 nM) resulted in decreased levels of C/EBPβ at baseline, and further repression after TGF-β1 treatment (5 ng/ml, 24 h) (Figure 3c). Loss- or gain-of-function of miR-155 did not significantly alter the LIP/LAP ratio (Supplementary Figure S3c). Further analysis by qPCR showed that the expression levels of miR-155 were around 4.5-fold higher in NMuMG cells compared with EpH4 cells at baseline, and further increased in NMuMG cells upon TGF-β1 exposure (Figure 3d), as previously reported.^31^ In comparison, the expression level of C/EBPβ (all isoforms) was considerably higher in EpH4 cells compared with NMuMG cells (Figure 3e).

### Loss of C/EBPβ shifts the TGF-β response from growth inhibition to EMT

**Figure 4:**
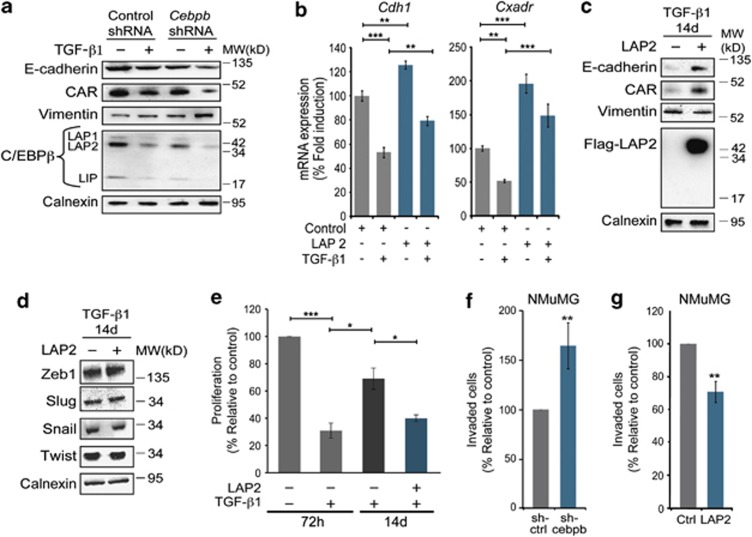
Loss of C/EBPβ shifts the TGF-β response from growth inhibition to EMT. (**a**) Immunoblotting results showing the effect of knockdown of C/EBPβ via overexpression of *Cebpb* shRNA (*Cebpb* shRNA) on the expression of EMT markers at baseline and after treatment with TGF-β1 (10 ng/ml, 48 h). Non-targeting shRNA was used as a control (Control shRNA). (**b**) qPCR results showing the effect of transient overexpression of LAP2 on *Cdh1* and *Cxadr* expression in NMuMG cells at baseline, and after TGF-β1 treatment (2 ng/ml, 24 h). Data from qPCR experiments (**f**, **g**) represent mean±s.e.m. of three independent experiments. *P<0.05, **P<0.01, ***P<0.001. (**c**, **d**) Immunoblotting results showing the effect of overexpression of FLAG-tagged LAP2 (FLAG-LAP2) on the expression of EMT markers in NMuMG cells treated for long term (14 days) with TGF-β1 (2 ng/ml). (**e**) Bar graph showing the effect of TGF-β1 treatment for 72 h (10 ng/ml) or 14 days (2 ng/ml) on the proliferation of NMuMG cells in the presence, or absence of overexpressed LAP2. Data represent mean±s.e.m. of three independent experiments. *P<0.05, ***P<0.001. (**f**, **g**) Bar graphs showing the effect of C/EBPβ knockdown through expression of *Cebpb* shRNA (**f**) or overexpression of LAP2 (**g**) on the capacity of NMuMG cells to migrate through Matrigel during 8 h using TGF-β1 (10 ng/ml) as a chemoattractant. Data are presented as percentage values compared with controls that were set to 100. *P<0.05, **P<0.01, ***P<0.001.

The data suggested that miR-155-mediated repression C/EBPβ could sensitize cells to TGF-β1-induced EMT. To study this, we performed loss- and gain-of-function studies for C/EBPβ. Knockdown of C/EBPβ in NMuMG cells through lentivirus-mediated overexpression of *Cebpb* small hairpin RNA (shRNA) resulted in decreased expression of E-cadherin and CAR, at baseline (Figure 4a). Furthermore, *Cebpb* knockdown potentiated induction of EMT in response to TGF-β1 (5 ng/ml, 24 h), as evident by further decreased expression of E-cadherin and CAR, and further increased expression of vimentin compared with cells expressing control shRNA (Figure 4a). On the contrary, transient overexpression of LAP2 in NMuMG cells resulted in increased mRNA levels of *Cdh1* and *Cxadr* at baseline, and less repression of both genes in response to TGF-β1 (2 ng/ml, 24 h) (Figure 4b). However, no rescue of E-cadherin, or CAR repression was detected at the protein level (data not shown), under these conditions.

Together, these results suggested that loss of C/EBPβ had an impact on the induction phase allowing NMuMG cells to more easily undergo EMT in response to TGF-β1. Next, we wanted to determine whether re-expression of C/EBPβ would be sufficient to revert cells from a more established EMT phenotype. To test this, we used a strategy of long-term induction of EMT in NMuMG cells, which previously was reported to result in profound EMT and also, evasion of the cytostatic effects of TGF-β1.^32^ We found that NMuMG cells treated for long term (14 days) with TGF-β1 (2 ng/ml) displayed a prominent EMT phenotype with low expression of E-cadherin, CAR and C/EBPβ (Figure 4c). Overexpression of LAP2 in such long-term treated NMuMG cells resulted in increased expression of E-cadherin and CAR, and decreased expression of vimentin. However, no morphological evidence of reversion of the EMT phenotype was observed (data not shown). Overexpression of LAP2 did not affect the expression of the EMT factors Zeb1, Slug, Snail or Twist, suggesting that the induction of E-cadherin and CAR was independent of the expression of these factors (Figure 4d). Similar to a previous report,^32^ we found that long-term treated NMuMG cells had evaded most of the growth inhibitory effect of TGF-β1 and proliferated at a rate more similar to untreated cells (Figure 4e). Overexpression of LAP2 was sufficient to restore the growth inhibitory effect of TGF-β1 in these cells (Figure 4e).

Together, these data indicated that miR-155-mediated depletion of C/EBPβ allows mammary tumor cells to both evade the cytostatic effect of TGF-β1, and become sensitive to TGF-β-induced EMT. Based on this, we hypothesized that C/EBPβ levels might also affect the invasive properties of NMuMG cells in response to TGF-β1. To test this, we performed two-chamber chemoinvasion assays, in which NMuMG cells expressing control shRNA, or *Cebpb* shRNA, were seeded in Matrigel in the upper chamber and TGF-β1 (10 ng/ml) added to the lower chamber. Analysis was performed at 8 h after seeding cells and showed that NMuMG cells expressing *Cebpb* shRNA were 60% more invasive than cells expressing control shRNA (Figure 4f). On the contrary, NMuMG cells overexpressing LAP2 were 40% less invasive compared with control cells (Figure 4g).

### Loss of C/EBPβ promotes invasion and metastatic spread of mouse mammary 4T1 tumor cells

**Figure 5:**
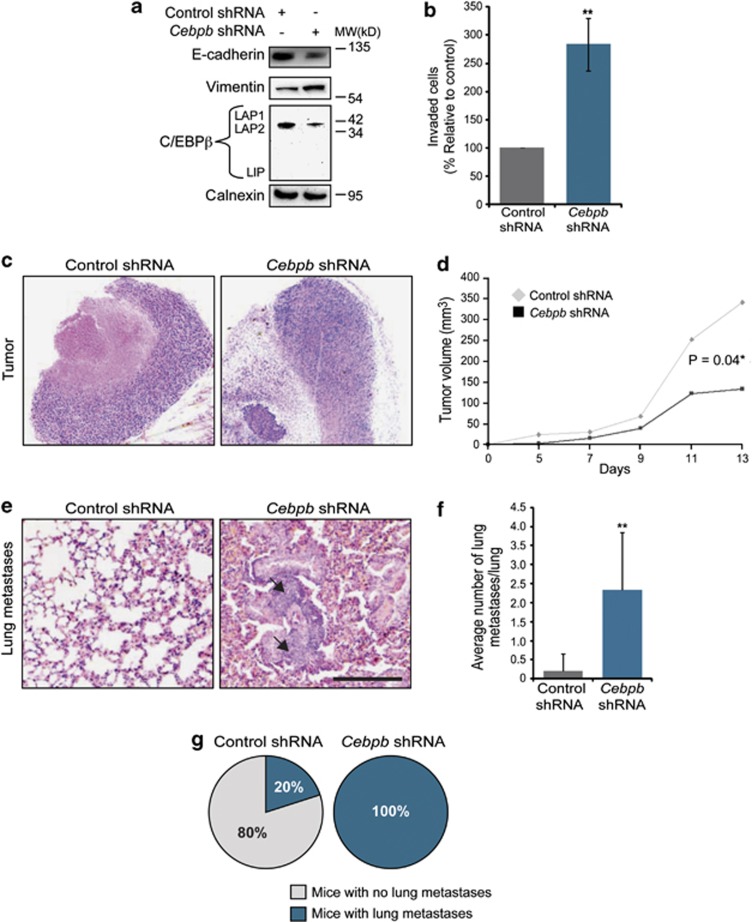
Loss of C/EBPβ promotes invasion and metastatic spread of mammary tumor cells. (**a**) Immunoblotting results showing the effect of overexpression of *Cebpb* shRNA on the expression of E-cadherin, vimentin and C/EBPβ in 4T1 cells. Calnexin was used as a loading control. (**b**) Bar graph showing the effect of C/EBPβ knockdown through expression of *Cebpb* shRNA on the capacity of 4T1 cells to migrate through Matrigel using TGF-β1 (10 ng/ml) as a chemoattractant. Data are presented as percentage values compared with controls that were set to 100. **P<0.01. (**c**) Representative H&E images of 4T1 tumors expressing control or *Cebpb* shRNA and grown subcutaneously (sc) for 2 weeks. (**d**) Bar graph showing growth rates of 4T1 tumors expressing control or *Cebpb* shRNA and grown sc for 2 weeks. (**e**) Representative H&E images of lung metastasis (arrows) from mice carrying 4T1 tumor cells expressing control or *Cebpb* shRNA. Scale bar, 200 μm (**c**, **e**). (**f**) Circle diagram showing percentage of mice with detectable lung metastases. (**g**) Bar graph showing the average number of lung metastasis. **P<0.01.

The results showing that C/EBPβ levels modified the invasive capacity of NMuMG cells towards TGF-β1 *in vitro* prompted us to study whether loss of C/EBPβ would affect the metastatic capacity of mammary tumor cells, *in vivo*. We performed loss-of-function studies in the 4T1 mammary tumor model, which is driven by TGF-β.^33^ Knockdown of C/EBPβ in 4T1 cells through overexpression of *Cebpb* shRNA resulted in decreased expression of E-cadherin, and increased expression of vimentin (Figure 5a). Similar to the results from the experiments with NMuMG cells, we also found that 4T1 cells expressing *Cebpb* shRNA were more invasive than control cells towards TGF-β1 (Figure 5b). Upon subcutaneous injection of 4T1 cells into the flank of syngeneic BALB/c mice, we found that 4T1 cells expressing *Cebpb* shRNA initially grew at a similar rate to control tumors but during the second week, their growth rate decreased (Figures 5c and d). Analysis of metastatic spread to the lungs showed that the average number of metastasis per lung was significantly higher in mice carrying C/EBPβ knockdown tumors (*N*=7) compared with mice carrying control tumors (*N*=5) (Figures 5e and f). Metastatic foci in the lungs were found in 100% of mice with C/EBPβ knockdown tumors and 20% of mice with control tumors (Figure 5g). Further analysis showed that knockdown of C/EBPβ did not affect phosphorylation of Smad3 after exposure to TGF-β1, or endogenous production of TGF-β1 in 4T1 cells (Supplementary Figures 4a and b). These results indicated that the effect of C/EBPβ knockdown was not due to altered TGF-β signaling in these cells. Furthermore, we were interested to study whether it would be possible to revert to EMT and inhibit the invasive capacity of 4T1 cells by stable overexpression of C/EBPβ. qPCR analysis revealed that 4T1 cells, similar to NMuMG cells, expressed high levels of miR-155 compared with EpH4 cells (Supplementary Figure 4c). Lentivirus-mediated overexpression of C/EBPβ did not affect the expression of EMT markers in 4T1 cells and also, did not affect the capacity of these cells to migrate in invasion assays (Supplementary Figures 4d and e). These results indicate that overexpression of C/EBPβ is not sufficient to revert tumor cells from a more stable EMT phenotype.

### Identification of C/EBPβ as a transcriptional activator of junction proteins

Based on our results, we wanted to elucidate the mechanisms by which loss of C/EBPβ promotes TGF-β1-induced EMT, invasion and metastasis. As our studies had shown that knockdown of C/EBPβ, *per se*, was sufficient to cause reduced expression of E-cadherin and CAR, we hypothesized that C/EBPβ could modulate the EMT response downstream of TGF-β by acting as a transcriptional activator of junction proteins.

**Figure 6:**
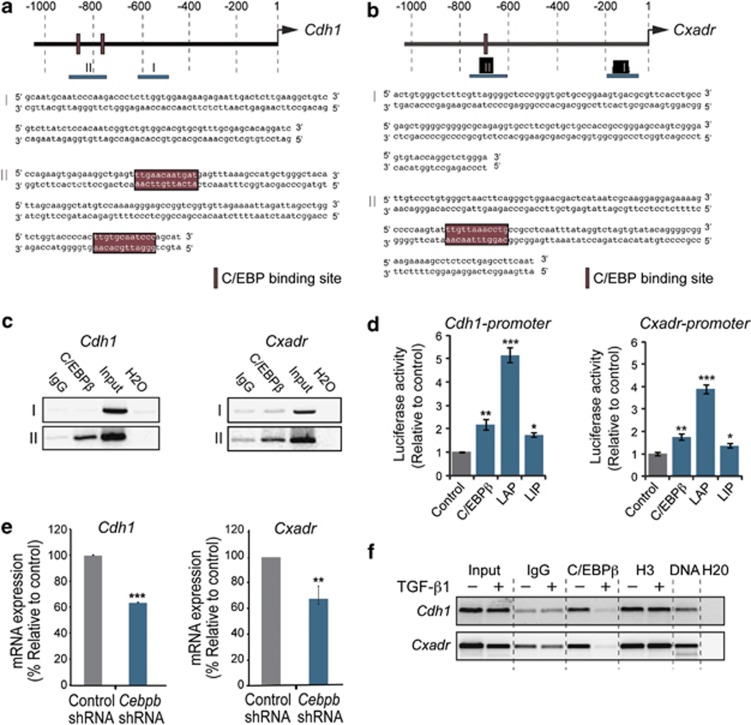
Identification of C/EBPβ is a transcriptional activator of genes encoding junction proteins in mammary epithelial cells. (**a**, **b**) Putative C/EBP binding sites in the promoter regions of the mouse *Cdh1* (E-cadherin) and *Cxadr* (CAR) genes were identified via the Consite software (http://asp.ii.uib.no:8090/cgi-bin/CONSITE/consite/). Regions containing (region II), or not containing (region I) C/EBP binding sites are shown. (**c**) Results from ChIP analysis of C/EBPβ binding to regions I/II in *Cdh1* and *Cxadr* promoters in NMuMG cells. (**d**) Results from promoter reporter assays showing the effect of overexpression of C/EBPβ, LAP2 or LIP on the activity of *Cdh1* and *Cxadr* promoters in EpH4 cells. Data represent mean ±s.d. *P<0.05, compared with control. (**e**) qPCR results showing the effect of *Cebpb* shRNA and control shRNA on *Cdh1* and *Cxadr* expression. (**f**) Results from ChIP analysis showing the effect of TGF-β1 (10 ng/ml, 48 h) on C/EBPβ binding to *Cdh1* and *Cxadr* promoters. Data from qPCR experiments represent mean±s.e.m. of three independent experiments. *P<0.05, **P<0.01, ***P<0.001.

By using a computer-based software used to identify binding sites for transcription factors in genomic DNA sequences, we found putative binding sites for C/EBP transcription factors were identified in promoter regions of several genes encoding junction proteins including *Cdh1* (Figure 6a), *Cxadr* (Figure 6b), *Cldn3* (Supplementary Figure 3a) and *Ocln* (Supplementary Figure 3b). Chromatin immunoprecipitationChIP assays demonstrated that C/EBPβ specifically interacted with regions of *Cdh1* and *Cxadr* promoters containing C/EBP binding sites in NMuMG cells (Figure 6c). To determine the capacity of C/EBPβ to regulate *Cdh1* and *Cxadr* promoters, we performed promoter reporter assays. Overexpression of C/EBPβ activated the *Cdh1* promoter by twofold and the *Cxadr* promoter by 2.5-fold (Figure 6d). Similarly, C/EBPβ activated *Ocln* and *Cldn3* promoters by fivefold and twofold, respectively (Supplementary Figure 3c). LAP2 was more potent than C/EBPβ and activated the *Cdh1* and *Cxadr* promoters by five- and fourfold, respectively. LIP had modest effects on *Cdh1* and *Cxadr* promoters.

In line with a role for C/EBPβ as a factor, which is important to maintain cellular levels of E-cadherin and CAR, we found that knockdown of C/EBPβ resulted in decreased *Cdh1* and *Cxadr* mRNA levels (Figure 6e). Knockdown of C/EBPβ resulted in loss of E-cadherin and CAR also in human lung A549 carcinoma cells suggesting a role for C/EBPβ as a transcriptional activator of junction proteins also in other types of epithelial cells (Supplementary Figure 3d). Furthermore, we found that C/EBPβ dissociated from *Cdh1* and *Cxadr* promoters during TGF-β1-induced EMT (Figure 6f).

## Discussion

**Figure 7:**
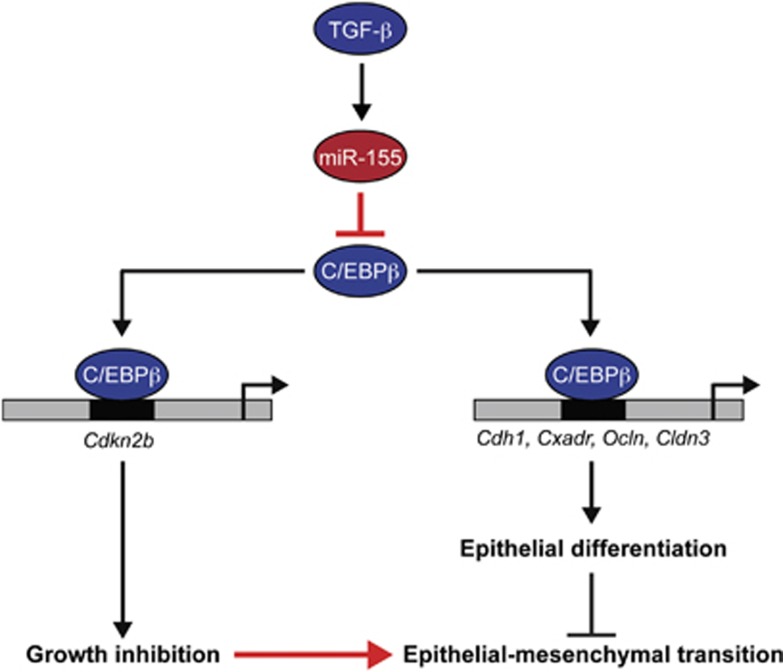
Schematic model proposing how miR-155-mediated depletion of C/EBPβ promotes breast cancer progression by shifting the TGF-β response from growth inhibition to EMT. C/EBPβ acts as a mediator of TGF-β-induced growth inhibition by acting as a transcriptional activator of *Cdkn2b*, which encodes p15INK4b, as shown by others.^18^ In addition, C/EBPβ promotes epithelial differentiation by acting as a transcriptional activator of genes encoding junction proteins including *Cdh1*, *Cxadr*, *Ocln* and *Cldn3*. Activation of miR-155, an oncomiR frequently overexpressed in breast cancer, results in depletion C/EBPβ. As a result, the growth inhibitory effect of TGF-β is lost, and as a consequence of inactivation of genes encoding junction proteins, cells acquire an increased capacity to undergo EMT, invade and metastasize.

Based on our results, we propose a model that miR-155-mediated depletion of C/EBPβ in breast cancer cells promotes loss of differentiation, invasion and metastasis by shifting the TGF-β response from growth inhibition to EMT (Figure 7). A mechanism explaining why loss of C/EBPβ promotes EMT, invasion and metastasis was traced to a previously uncharacterized role of C/EBPβ as a transcriptional activator of junction proteins in mammary epithelial cells. Thus, loss of C/EBPβ-mediated gene activation of *Cdkn2b*, as well as *Cdh1* and *Cxadr*, shifts the TGF-β response in breast cancer cells from growth inhibition to EMT.

Loss of C/EBPβ was detected in samples of triple-negative human breast cancers, which are characterized by their de-differentiated and invasive nature, and by displaying EMT characteristics, such as loss of E-cadherin.^8^ This raises the possibility to use staining for C/EBPβ as an additional diagnostic tool to determine invasiveness in human breast cancer. In support of this, reduced staining of C/EBPβ is seen in various samples of ductal breast carcinomas presented in the Human Protein Atlas database.^34^ Future studies using a more large-scale approach will determine whether immunohistochemical staining of C/EBPβ could be used as prognostic tool in breast cancer.

The identification of C/EBPβ as a transcriptional activator of junction proteins, not only in mammary epithelial cells but also in human lung carcinoma cells, provides a mechanism for how loss of C/EBPβ promotes EMT, tumor cell invasion and metastasis. The results should be put into perspective of published data showing that epithelial genes including *Cdh1* and *Cxadr*, are inactivated during EMT by repressors, such as Snail, Zeb and Twist (reviewed in Peinado *et al.*^35^). Our results suggest that inactivation of epithelial genes during EMT is not solely a result from activation of transcriptional repressors, but also from inactivation of transcriptional activators, such as C/EBPβ, which normally promote epithelial differentiation. In our studies, it was not sufficient to revert EMT in 4T1 tumor cells by stable expression of C/EBPβ alone. It is possible that C/EBPβ needs to be overexpressed together with other cofactors to fully induce mesenchymal-epithelial transition in invasive cancer cells. It might also be due by incapacity of C/EBPβ to overcome the repressive action of Snail, Zeb and Twist factors on epithelial genes. It will be of interest for the future to elucidate whether activators and repressors compete to regulate epithelial genes and whether such competition would be important for mammary epithelial cell differentiation, and for the induction of EMT during breast cancer progression. Identification of such mechanisms may open up possibilities to block or even revert EMT to inhibit tumor progression. Interestingly, vimentin was more potently induced by TGF-β after knockdown of C/EBPβ, and further repressed after overexpression of LAP2. This indicates that C/EBPβ may promote epithelial differentiation both by activating epithelial genes and by repressing mesenchymal genes. Future studies will reveal whether C/EBPβ is a direct repressor of vimentin, or if it acts through one or more intermediate steps of regulation.

We identified C/EBPβ as a target of miR-155 during TGF-β-induced EMT in NMuMG cells. MiR-155 is an oncomiR linked to breast cancer invasion and metastasis and was recently shown to be an important mediator of TGF-β-induced EMT in NMuMG cells.^31, 36^ However, few miR-155 targets have been linked to TGF-β-induced EMT. It was recently shown that miR-155 targets RhoA,^31^ which regulates formation of cell–cell junctions and cell polarity during epithelialization by controlling actomyosin polymerization and protein complex formation.^37, 38^ Together with our results, this suggests that miR-155 promotes degradation of junction complexes during TGF-β-induced EMT by targeting dual mechanisms: (i) RhoA-mediated actomyosin polymerization and junction formation, and (ii) C/EBPβ-mediated transcriptional activation of genes encoding junction proteins.

Re-activation of C/EBPβ and other factors promoting epithelial differentiation might represent a fruitful approach to preserve or strengthen a more differentiated, benign tumor phenotype in breast cancer. In support of this, it has been shown that steroid hormones like glucocorticoids, which are potent inducers of C/EBPβ and that promote epithelial differentiation, can inhibit TGF-β-induced EMT in breast cancer cells, and block EMT and tumor cell dissemination in pancreatic cancer models.^39, 40, 41^ In addition, C/EBPβ is a critical mediator of progesterone-regulated epithelial differentiation, and it was recently shown that progesterone reverses the EMT phenotype in breast cancer cells, and inhibits EMT in endometrial cancer.^42^ Moreover, BMP7, which was recently shown to antagonize TGF-β-induced EMT and inhibit metastatic spread of breast cancer cells, is a potent inducer of C/EBPβ, at least in adipocytes.^43, 44, 45^ Based on our data, we believe it would be of interest for the future to study the role of C/EBPβ in mediating the anti-EMT effects of these agents and also, to elucidate further target genes of C/EBPβ in breast cancer cells.

## Materials and methods

### Human breast cancer

Clinical samples of human ductal carcinoma *in situ* and invasive ductal breast cancer were obtained after breast cancer surgery and were freshly frozen and sectioned according to standard procedures. A pathologist at Umeå University Hospital, Sweden independently classified all tumors according to the status of estrogen, progesterone and HER2 receptor. The Research Ethics Review Board of Northern Sweden (EPN) approved all studies using human material.

### Mouse mammary tumor models

All animal studies were approved by the Northern Stockholm Animal Welfare Committee, and by the Karolinska Institute. Mammary tumors and lungs were isolated from 10- or 14-week-old female heterozygous MMTV-PyMT mice (Jackson Laboratories, Bar Harbor, ME, USA), freshly frozen and sectioned according to standard procedures. Mammary tumors were harvested at 10 weeks of age to 14 weeks of age. For frozen sections, samples were fixed 1 h at 4 °C in 4% paraformaldehyde, incubated in 30% sucrose over night, embedded in OTC, sectioned and stained as indicated.

Green fluorescent protein-labeled 4T1 cells (2 × 10^6^) expressing *Cebpb* shRNA or control shRNA were subcutaneously implanted in the dorsal back of 6- to 8-week-old female BALB/c mice (*n*=8 per group) and tumor sizes were measured with a caliper every 2 days, starting from day 5 after implantation. To study metastasis, primary tumors were surgically removed under analgesia and anesthesia 2 weeks after implantation. Animals were killed after 2 months and their lungs isolated, fixed in 4% paraformaldehyde and embedded in paraffin. Central parts of lungs from each mouse implanted with 4T1 cells either expressing *Cebpb* shRNA (*N*=7) or control shRNA (*N*=5) were sectioned (5 μm), stained for hematoxylin and eosin and analyzed with a Leica DMLB100 bright field microscope at × 10 and × 40 magnification to morphologically identify metastases. Pictures were captured at × 20 magnification.

### Cell culture and treatments

NMuMG cells (ATCC) were cultured as described previously.^11^ EMT was induced in by the addition of TGF-β1 (R&D Systems, Abingdon, UK) at doses of 2–10 ng/ml. EpH4 cells (kindly provided by H. Beug, Vienna Medical University) were cultivated as described.^46^ Human lung A549 carcinoma cells (ATCC) were cultivated in Dulbecco's modification of Eagle's medium with 10% fetal calf serum at 37 °C. Mouse mammary 4T1 tumor cells (ATCC) were cultured in RPMI-1640 with 10% fetal calf serum at 37 °C.

The specific inhibitor of Smad3 (Calbiochem, EMD Chemicals, Gibbstown, NJ, USA) was added to NMuMG cells (10 nM) 2 h prior to the addition of TGF-β1. After 24 h, cells were harvested and analyzed by immunoblotting. The Smad3 siRNA (Dharmacon, Thermo Scientific, Rockford, IL, USA), the miR-155 inhibitor (miRIDIAN Hairpin Inhibitor mmu-miR-155, Dharmacon, Thermo Scientific) and the miR-155 mimic (Syn-mmu-miR-155 miScript miRNA Mimic, Qiagen, Stockholm, Sweden), or the negative control (miRIDIAN microRNA hairpin inhibitor negative control no 1 (Dharmacon)) were transfected into NMuMG cells in 12-well plates using Dharmafect according to standard protocol 24 h prior to addition of TGF-β1 (5 ng/ml). Cells were lysed after 24 h and analyzed by immunoblotting. For qPCR analysis, cells were transfected in 24-wells.

### Immunofluorescence staining and image analysis

Immunofluorescence staining of cultured cells was performed on cells grown on cover slips, which fixed in absolute ethanol, and stained as previously described.^11^ Staining of frozen sections of human and mouse tumors was performed after fixation in 4% paraformaldehyde, blocking in 3% BSA in PBS followed by staining with primary and secondary antibodies according to Supplementary Methods.

For image analysis and quantification of the intensity of immunofluorescence staining in the eight different human invasive breast carcinomas, three to five images per tumor were captured ( × 200 magnification), depending on tumor size. Images were imported into the ImageJ software (http://rsbweb.nih.gov/ij/). Four randomly selected tumor areas in each image were analyzed separately for mean fluorescence intensity staining of C/EBPβ and E-Cadherin, respectively. To more specifically study whether loss of C/EBPβ correlated with loss of E-cadherin, we compared mean fluorescence intensity values in E-cadherin^+^ versus E-cadherin^−^ areas in the three tumors that were classified as triple-negative breast cancers. Four E-cadherin^+^ and four E-cadherin^−^ areas were selected per tumor. For quantification of mean fluorescence intensity of C/EBPβ and E-Cadherin staining in tumor sections from 14-week-old MMTV-PyMT mice (*N*=3) five images of each tumor were captured. Images were imported into ImageJ and four E-cadherin^+^ and four E-cadherin^−^ areas were selected per tumor and compared for C/EBPβ intensity.

### Antibodies

See Supplementary section.

### Lentivirus-mediated knockdown and overexpression of C/EBPβ

Lentivirus vectors expressing *shCebpb* or *control shRNA* (Santa Cruz Biotechnology, Santa cruz, CA, UA) were used to infect NMuMG, 4T1 and A549 cells. Vectors overexpressing human C/EBPβ or control (Gentarget, San Diego, Ca, USA) were used to infect 4T1 cells. Briefly, 1 × 10^5 cells were seeded in a 96-well plate and infected with 20 μl of lentivirus particles in medium containing 8 μg/μl polybrene (Calbiochem, EMD). After 24 h, medium was replaced with selection medium containing 10 μg/ul Puromycin (Calbiochem, EMD).

### Immunoblotting

Immunoblotting analysis of cell lysates from cells prepared at indicated times was performed according to standard procedures as previously described.^11^

### Quantitative real-time PCR

Was performed according to standard protocols as previously described.^11^ Primers used are listed in Supplementary Table 1.

### Reporter assays and overexpression studies

The expression vector for rat *Cebpb* was provided by Magnus Nord (Lung Research Laboratory, Karolinska Hospital, Sweden). Expression plasmids for human *LIP* and *LAP2* isoforms were obtained from Addgene, Cambridge, MA, USA. A promoter reporter construct for human *CDH1* was generated by subcloning a 2-kb sequence upstream region of *CDH1* from a fosmid containing a 10-kb genomic DNA sequence upstream from *CDH1* (BACPAC Resources Center, Oakland, CA, USA) into a pGL-3 basic vector (Promega, Fitchburg, WI, USA) by introducing the restriction sites Nhe1 and HindIII using PCR (for primers, see Table S1). Plasmids encoding human *Ocln* and mouse *Cldn3* promoters were provided by J. Mankertz (Charité - Universitätsmedizin Berlin, Germany) and M. Furuse (Kobe University, Japan), respectively. The mouse *Cxadr* promoter construct was described previously.^11^ A plasmid encoding *β-Galactosidase* (*β-gal*) under the *CMV* promoter was used as an internal control of transfection efficiency in all reporter assays.

Reporter constructs and expression plasmids were transfected into EpH4 or NMuMG cells using Lipofectamine 2000 (Invitrogen, Carlsbad CA, USA) according to standard procedures. Effects on mRNA expression were analyzed by qPCR as described. Luciferase and β-GAL activities were analyzed using a Polar Star Omega (BMG LABTECH, Cary, NC, USA) plate reader. Luciferase values were normalized to β-GAL values to account for variations in transfection efficiency.

### Invasion assays

Cells were trypsinized, resuspended in growth factor-reduced Matrigel (2 mg/ml, BD Biosciences, Stockholm, Sweden) diluted 1:5 in Dulbecco's modification of Eagle's medium with 1% FBS. Fifty-thousand cells were seeded into the cell culture inserts (8 μm pore (Millipore, Billerica, MA, USA). Medium with TGF-β1 (10 ng/ml) was added in the lower chamber. After 8-h incubation in 37 °C/ 5% CO_2_ incubator, non-migrated cells were removed with cotton swabs and inserts fixed in methanol. The membrane was removed and mounted with Vectashield containing 4',6-diamidino-2-phenylindole (Vector Labs, Burlingame, CA, USA). Images were captured by using a Nikon Eclipse E800 microscope and the number of invaded cells was counted.

### Chromatin immunoprecipitation assays

Chromatin immunoprecipitation assays were performed using a chromatin immunoprecipitation Assay Kit (Millipore), according to instructions from the manufacturer. DNA-protein complexes were amplified by PCR for 25–30 cycles in the presence of 1.5 mM MgCl_2_, 200 μM dNTPs and 10 pmol of primers (see Table S1). Ten percent of the precipitated DNA was used for each PCR. Samples were analyzed on a 2% agarose gel. All chromatin immunoprecipitation experiments were repeated three times.

### Statistical analysis

All values are presented as means±s.e.m. Significance between two groups was determined by *t*-test (reporter assays, qPCR) and significance as assessed by analysis of variance, followed by the Bonferroni's test for multiple comparisons. Linear regression analysis of mean fluorescence intensity values for C/EBPβ and E-Cadherin expression in human tumors was analyzed using PrismGraphPad. *P*<0.05 was considered significant for all statistical analysis.
