# Supplementary material for: MiR-155-mediated loss of C/EBPβ shifts the TGF-β response from growth inhibition to epithelial-mesenchymal transition, invasion and metastasis in breast cancer
Source: Oncogene. 2013 Aug 19;32(50):5614–24. doi: 10.1038/onc.2013.322 (PMC3898103; doi:10.1038/onc.2013.322)
Supplement: Supplementary Information [file onc2013322x1.pdf]

Oncogene

Supplemental Information

MiR-155-mediated loss of C/EBPβ shifts the TGF-β response from growth inhibition to epithelial-mesenchymal transition, invasion and metastasis in breast cancer

Joel Johansson[1,#], Tove Berg[1,#], Ewa Kurzejamska[2,4], Mei-Fong Pang[1], Vedrana Tabor[1], Malin Jansson[3], Pernilla Roswall[1], Kristian Pietras[1], Malin Sund[3], Piotr Religa[2] and Jonas Fuxe[1,§]

Inventory of Supplemental Information

Supplementary Figure 1

Supplementary Figure 2

Supplementary Figure 3

Supplementary Figure 4

Supplementary Figure 5

Supplementary Material and Methods

Tumor#	ER	PR	HER2
1	3	2	3
2	2	2	2
3	1	2	0
4	0	0	2
5	0	0	2
6	0	0	0
7	0	0	0
8	0	0	0

Expression
3 High
2
1
0 Neg

Supplementary Figure 1. Table showing characterization of prognostic markers in the 8 human invasive ductal breast cancers that were included in the study, as classified by a pathologist. ER, estrogen receptor; PR, progesterone receptor; HER2, human epidermal growth factor receptor.

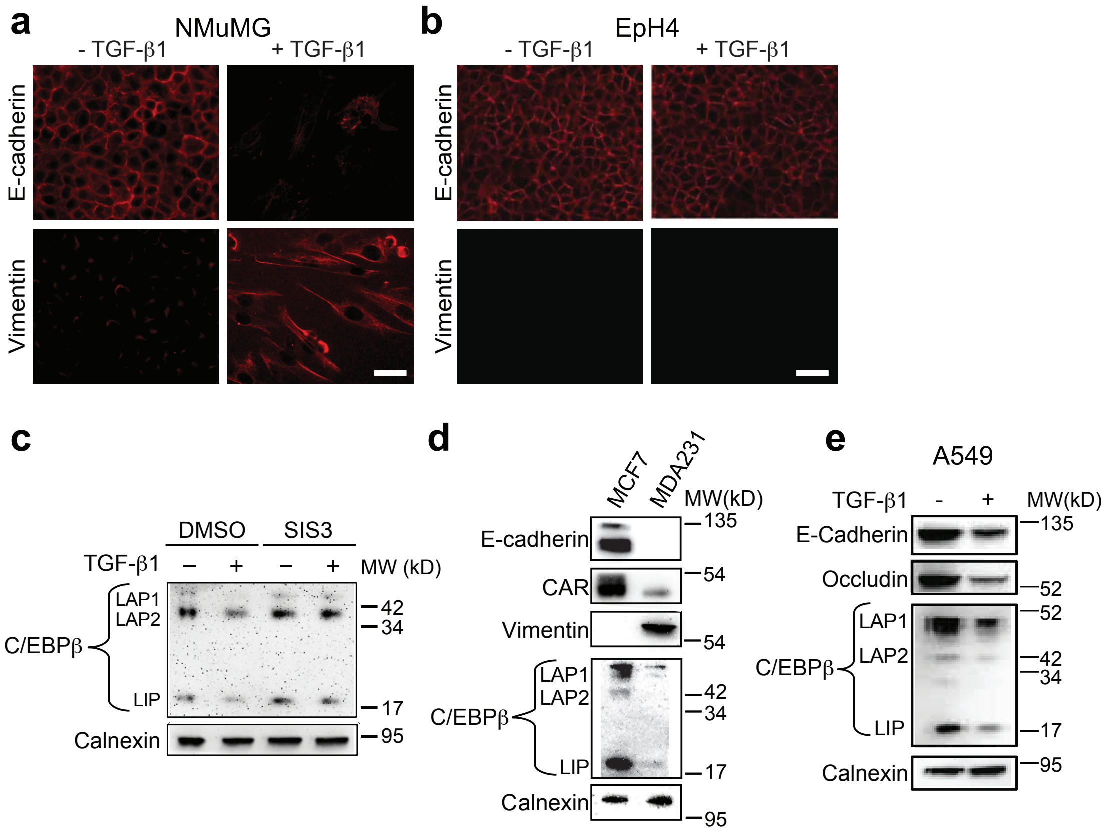

Supplementary Figure 2. Loss of C/EBPβ during EMT. (**a**, **b**) Treatment with TGF-β1 (10ng/ml for 48 h) results in the induction of EMT features including loss of E-cadherin, induction of vimentin and cellular elongation in NMuMG cells (**a**), but not in EpH4 cells (**b**). (**c**) Immunoblotting analysis of the effect of a specific inhibitor of Smad3 (SIS3, 10nM) on the repression of C/EBPβ during TGF-β1-induced EMT (5 ng/ml for 24 h) in NMuMG cells. (**d**) C/EBPβ is significantly less expressed in invasive MDA-MB-231 (MDA231) human breast cancer cells, which display EMT features, than in non-invasive MCF-7 human breast cancer cells, which dispaly a more differentiated phenotype. (**e**) Induction of EMT in human A549 lung carcinoma cells by TGF-β1 (10 ng/ml for 48 h) is associated with loss of C/EBPβ. Calnexin was used as a loading control for all immunoblotting experiments.

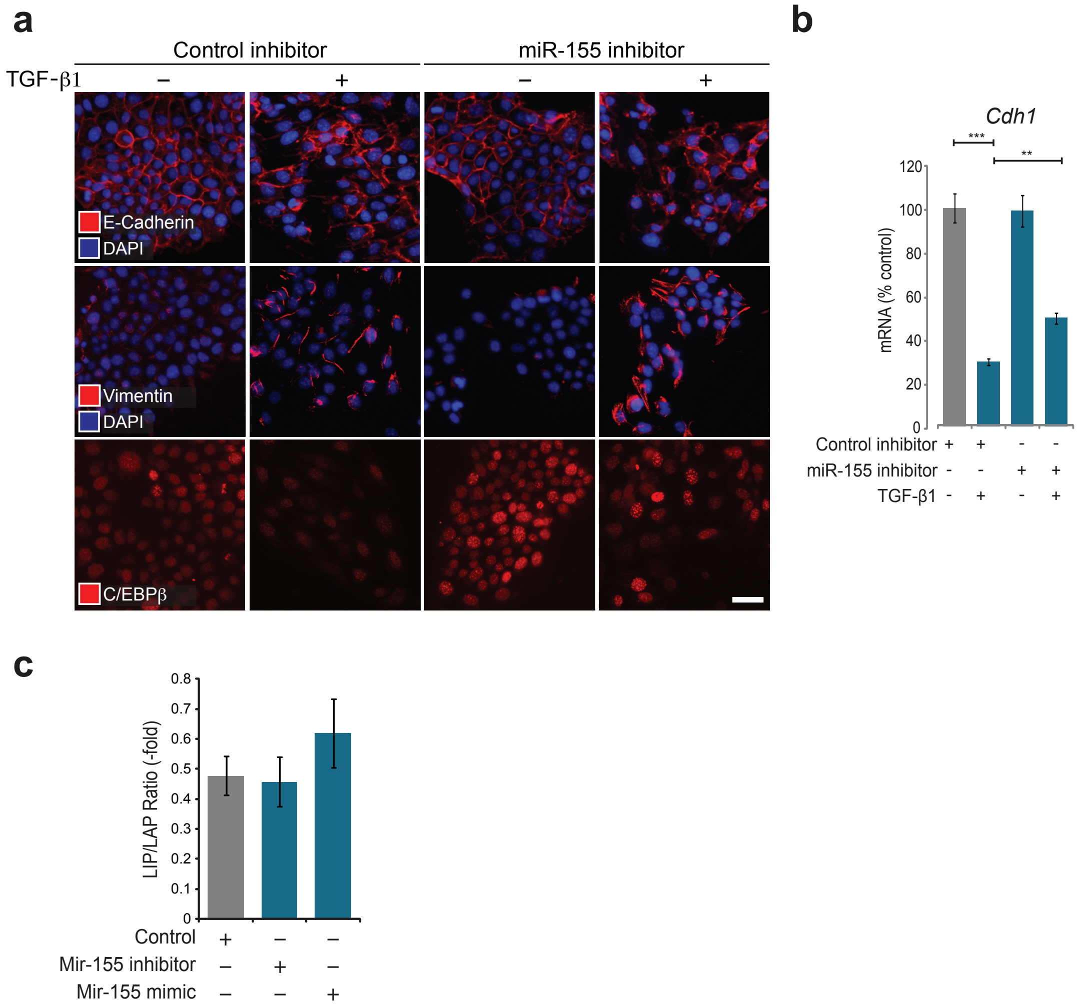

Supplementary Figure 3. (**a**) Immunofluorescence images showing the effect of a miR-155 inhibitor on the expression of E-cadherin, vimentin and C/EBPβ in NMuMG cells at baseline and after treatment with TGF-β1 (5ng/ml, 24h). (**b**) Bar graph showing he effect of a miR-155 inhibitor on the expression of Cdh1 mRNA at baseline and after treatment with TGF-b1 (5ng/ml, 24h).(**c**) Bar graph showing the effects of a miR-155 inhibitor and a miR-155 mimic (each one was used at a concentration of 50 nM and analysis was performed after 72 h) on the ratio between the LIP and LAP isoforms of C/EBPb in NMuMG cells..

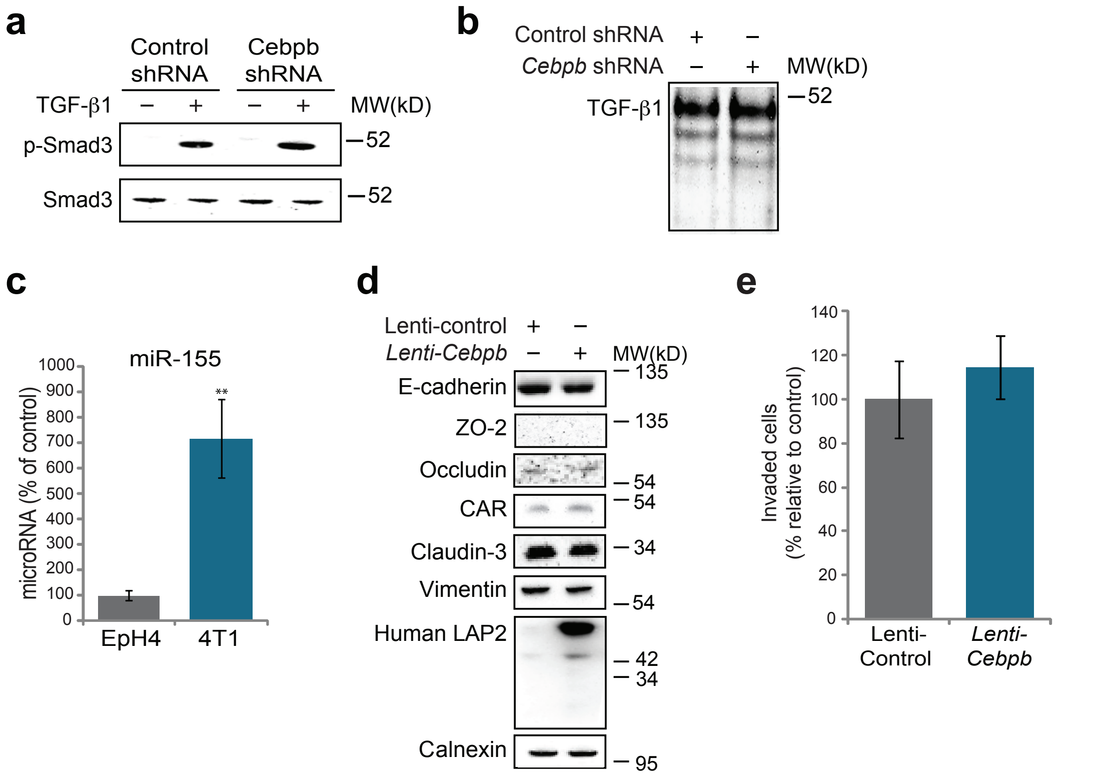

Supplementary Figure 4. Effects of manipulating C/EBPβ levels in 4T1 mammary tumor cells. (**a, b**) Results from immunoblotting analyses showing that C/EBPβ knockdown does not affect the TGF-β1 effect on Smad3 phosphorylation (a, p-Smad3) or on the endogenous expression of TGF-β1 (**b**). (**c**) Results from Q-PCR analysis showing significantly increased expression levels of miR-155 in 4T1 tumor cells compared to EpH4 cells. (**d**) Immunoblotting results showing that lentivirus-mediated overexpression of C/EBPβ does not lead to the reversion of EMT in 4T1 tumor cells. (**e**) Bar graph showing that overexpression of C/EBPβ in 4T1 cells does not have an impact of the capacity of these cells to invade and migrate in invasion assays.

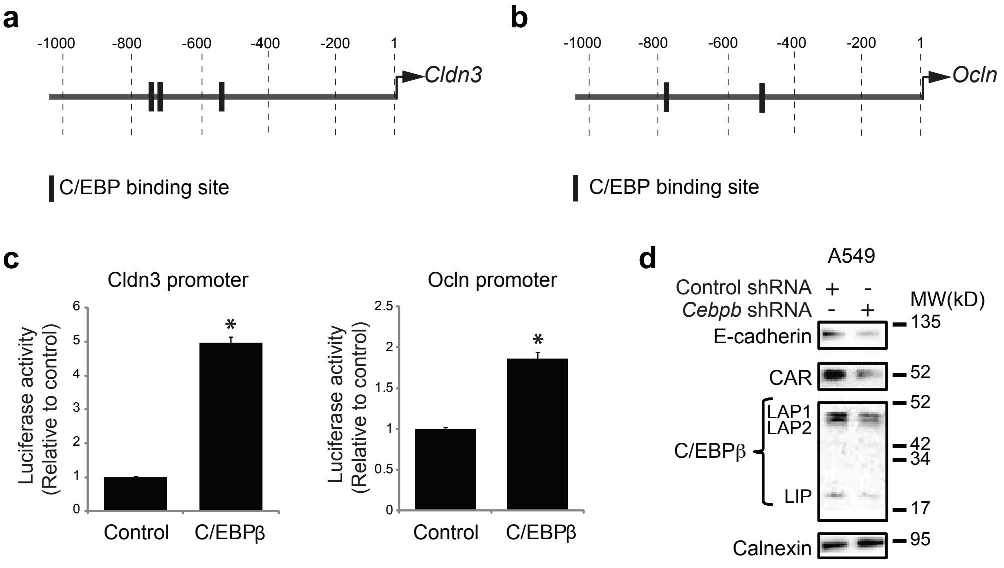

Supplementary Figure 5. Role of C/EBPβ as a transciptional activator of genes encoding junction proteins. (**a**, **b**) Putative C/EBP binding sites were identified via the Consite software (see method section) in gene promoters of both mouse Cldn3 (**a**) and Ocln (**b**). (**c**) Promoter reporter assays showing the capacity of C/EBPβ to activate expression of the luciferase reporter, which was cloned after the Cldn3 and Ocln promoters in pGL3 vectors that were co-transfected with the C/EBPβ expression plasmid into EpH4 cells. (d) Immunoblotting results showing the effect of C/EBPβ knockdown via transient overexpression of *Cebpb* shRNA on the expression of E-cadherin, CAR and C/EBPβ isoforms in human A549 lung carcinoma cells. Calnexin was used as a loading control.

Supplementary Material and methods

Antibodies

The following antibodies were used for immunostaining of cultured cells, and human and mouse tissue specimens: rabbit α-CAR (1:500) (43), mouse α-E-cadherin (1:200-1:500; clone 36, BD Biosciences, San Jose, CA), rabbit α-C/EBPβ sc-150 (1:100, Santa Cruz Biotechnology, Santa Cruz, CA), mouse α-Vimentin (1:1000, 3B4, DAKO, Stockholm, Sweden), and N-cadherin (1:100, ab12221, Abcam, Cambridge, UK). Alexa Fluor-conjugated secondary antibodies were used (1:200-1:1000, Jackson ImmunoResearch, Suffolk, UK).

Antibodies used for immunoblotting were rabbit α-CAR (1:500) (43), mouse α-E-cadherin (1:1000; clone 36, BD Biosciences, San Jose, CA), rabbit α-C/EBPβ sc-150 (1:500, Santa Cruz Biotechnology, Santa Cruz, CA), mouse α-Vimentin (1:1000, 3B4, DAKO, Stockholm, Sweden) rabbit α-Calnexin (1:2000) (43), N-cadherin (1:500, ab12221, Abcam, Cambridge, UK), rabbit anti-claudin-3 (1:500; 34-1700, Invitrogen, CA, USA), mouse anti-Snail (supernatant, 1:10, kind gift from Ismo Virtanen, Helsinki University), rabbit anti-Slug (1:500, clone C19G7, Cell signaling), rabbit anti-Zeb1 (1:250, NBP1-05987, Novus Biologicals, Stockholm, Sweden), mouse anti-Twist (1:500, sc-81417, Santa Cruz), rabbit anti-phospho-SMAD3 (1:500, C25A9, Cell signaling, MA, USA), mouse anti-Occludin, 611090, BD Bioscience, CA, USA), and α-mouse and α-rabbit Horseradishperoxidase IgG (1:8000, #7074, Cell Signaling Technology, Danvers, MA).

Antibodies used for Chip were Chip 4μg of rabbit α-C/EBPβ sc-150 (1:100, Santa Cruz Biotechnology, Santa Cruz, CA), 4μg of Histone H3, (ab1791) and rabbit IgG (ab46540) both from Abcam, Cambridge, UK.

Supplementary Table 1. List of primers used.

**Primer name**	**Sequence 5´-3**	**Use**
Cdh1 forward:	GACCGCTAGCCATAGCAAGGCTATGTC	*Cdh1* promoter
Cdh1 reverse:	GACCAAGCTTAGCAAACACTGAGCTCG	*Cdh1* promoter
Cdh1	QT00121163*	Q-PCR
Cxadr	QT00132489*	Q-PCR
Gapdh	QT00309099*	Q-PCR
Rpl19	QT01779218*	Q-PCR
Vim	QT00159670*	Q-PCR
Cdh1 (147bp) forward II	CCAGAAGTGAGAAGGCTGAG	Q-PCR
Cdh1 (147bp) reverse II	ATGCTGGGATTGCACAAG	ChIP
Cdh1 (109bp) forward I	GCAATGCAATCCCAAGAC	ChIP
Cdh1 (109bp) reverse I	GATCCTGTGCTCGCAAAC	ChIP
Cxadr (136bp) forward I	AACACTGTGGGCTCTTGCTT	ChIP

Cxadr (136bp) reverse I	TCCCAGAGCCTGGTACACTC	ChIP
Cxadr (145bp) forward II	TTGTCCCTGTGGGCTAAC	ChIP
Cxadr (145bp) reverse II	ATTGAAGGCTCAGGAGAGG	ChIP

*Product number of validated primers obtained from Qiagen
